# Supplementary material for: FD-2, an Anticervical Stenosis Device for Patients Undergoing Radical Trachelectomy or Cervical Conization
Source: Bioengineering (Basel). 2023 Sep 1;10(9):1032. doi: 10.3390/bioengineering10091032 (PMC10525620; doi:10.3390/bioengineering10091032)
Supplement: Supplementary file 1 [file bioengineering-10-01032-s001.zip › bioengineering-2455693-supplementary.pdf]

bioengineering

Brief Report

FD-2, an Anticervical Stenosis Device for Patients Undergoing Radical Trachelectomy or Cervical Conization

Seiji Mabuchi [1,*], Shoji Kamiura [1], Takuya Saito [2], Hayato Furukawa [2], Azusa Abe [3] and Takashi Sasagawa [4]

[1] Department of Gynecology, Osaka International Cancer Institute, 3-1-69, Otemae, Chuo-ku, Osaka-shi 541-8567, Japan; kamiura-sh@oici.jp
[2] Medical Technical Sec., Fuji Latex Co., Ltd., 1705 Chizuka-machi, Tochigi 328-0135, Japan; t-saitou@fujilatex.jp (T.S.); ha-furukawa@fujilatex.jp (H.F.)
[3] Quality Control Sec., Fuji Latex Co., Ltd., 1705 Chizuka-machi, Tochigi 328-0135, Japan; a-tanifuji@fujilatex.jp
[4] Fuji Latex Co., Ltd., 3-19-1 Kanda Nishiki-cho, Chiyoda-ku, Tokyo 101-0054, Japan; sasagawa@fujilatex.jp
* Correspondence: seiji.mabuchi@oici.jp; Tel.: +81-6-6945-1181

Supplementary

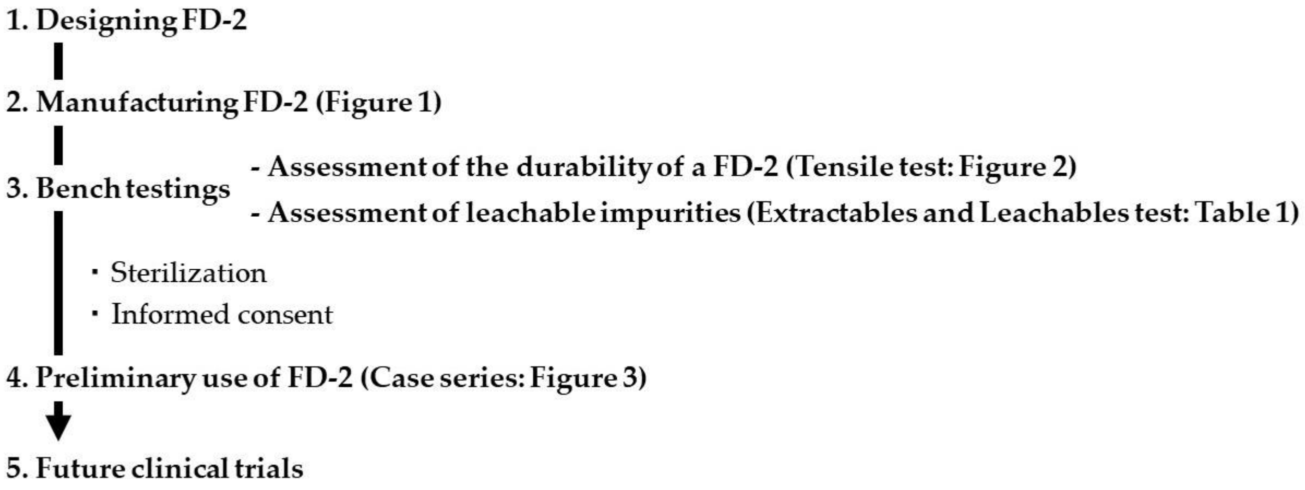

Figure S1. Flow diagram of the FD-2 development
